# Early Reciprocal Effects in a Murine Model of Traumatic Brain Injury and Femoral Fracture

**DOI:** 10.1155/2021/8835730

**Published:** 2021-01-18

**Authors:** Katharina Ritter, Kirsten Jung, Christopher Dolderer, Dominik Appel, Christine C. Oswald, Ulrike Ritz, Michael K. E. Schäfer

**Affiliations:** ^1^Department of Anesthesiology, University Medical Center of the Johannes-Gutenberg-University, Mainz, Germany; ^2^Department of Orthopedics and Traumatology, University Medical Center of the Johannes-Gutenberg-University, Mainz, Germany; ^3^Research Center for Immunotherapy (FZI), Germany; ^4^Focus Program Translational Neurosciences (FTN), Germany

## Abstract

Traumatic brain injury (TBI) represents a major cause of death and disability in early adulthood. Concomitant extracranial injury such as long bone fracture was reported to exacerbate TBI pathology. However, early reciprocal effects and mechanisms have been barely investigated. To address this issue, C57BL/6N mice were subjected to either the controlled cortical impact (CCI) model of TBI, fracture of the left femur (FF), combined injury (CCI+FF), or sham procedure. Behavioral alterations were monitored until 5 days post injury (dpi), followed by (immuno-)histology, gene and protein expression analyses using quantitative PCR, western blot, and ELISA. We found that CCI+FF mice exhibited increased neurological impairments, reduced recovery, and altered anxiety-related behavior compared to single injury groups. At 5 dpi, cerebral lesion size was not affected by combined injury but exaggerated hippocampal substance loss and increased perilesional astrogliosis were observed in CCI+FF mice compared to isolated CCI. Bone gene expression of the osteogenic markers Runx2, osteocalcin, alkaline phosphatase, and bone sialoprotein was induced by fracture injury but attenuated by concomitant TBI. Plasma concentrations of the biomarkers osteopontin and progranulin were elevated in CCI+FF mice compared to other experimental groups. Taken together, using a murine model of TBI and femoral fracture, we report early reciprocal impairments of brain tissue maintenance, behavioral recovery, and bone repair gene expression. Increased circulating levels of the biomarkers osteopontin and progranulin indicate ongoing tissue inflammation and repair. Our results may have implications for future therapeutic approaches to interfere with the pathological crosstalk between TBI and concomitant bone fracture.

## 1. Introduction

Traumatic brain injury (TBI) is one of the leading causes of death and lifelong disabilities in early adulthood and represents a severe medical and socioeconomic burden worldwide [1, 2]. As it frequently occurs in consequence of road accidents and falls, TBI is commonly accompanied by one or more potentially life-threatening, concomitant injuries [3]. In return, multiple trauma, defined as the presence of two or more separate injuries, with at least one or several of them in combination endangering the patient's life, often includes moderate and severe head injuries, which contribute significantly to mortality in those patients [4–6]. Injuries of extremities including fractures and expanded soft-tissue disruption are, together with chest and spine traumata, the most frequently observed companions of TBI in patients suffering from severe multiple trauma [4, 7]. As TBI globally still causes an age-standardized rate of 111 years lived with disability (YLDs) per 100,000 population, exploration of parameters contributing to neurological outcome and identification of potential therapeutic targets are major research subjects [1]. The reciprocal effects of TBI and concomitant bone fracture represent a potential cofactor of great clinical significance. However, interactions between the two injuries are poorly understood in the clinical and experimental settings and more insights into the pathophysiological mechanisms are needed [4, 8].

Injury after TBI is subdivided in primary and secondary brain damage comprising the initial impact of the trauma and the mechanical forces directly acting on the brain tissue. Secondary processes include cerebral ischemia, edema, mitochondrial dysfunction, necrosis and apoptosis, and inflammatory cascades involving a myriad of endogenous factors [9–11]. Bone fracture likewise causes local and systemic inflammatory responses, and subsequent processes of bone healing are intertwined with those of acute inflammation and innate immune system [12]. While the early postfractural phase is dominated by hematoma organization and an overall proinflammatory environment, subsequent transformation of soft fibrocartilage callus into the hard mineralized callus requires a versatile and long-term interaction of various skeletal cell types and growth factors [13].

Existing data on the interactions between TBI and bone fracture focus mainly on osseous remodeling effects and pay less attention to cerebral processes. Animal models of combined injury demonstrated accelerated bone healing and enlarged callus formation in fractures accompanied by TBI [14–17]. These findings are in agreement with clinical data showing increased callus volumes in patients with concomitant TBI and heterotopic ossifications in patients with sole TBI [13, 18, 19].

The consequences of concomitant bone fracture on secondary brain damage are less well elucidated, but exacerbated cerebral edema and neuroinflammatory responses including increased astrogliosis and behavioral impairments were reported in murine models of combined injury [8, 20, 21]. However, conclusive data from early posttraumatic time points are still scarce. The objective of this study was to investigate the early reciprocal interactions between TBI and a concomitant long bone fracture. Behavioral, (immuno-)histological, and protein and gene expression analyses were performed at 5 days post injury (dpi) to capture the transition phase from an acute inflammatory reaction to a subacute phase of remodeling and repair.

## 2. Methods

### 2.1. Animals and Study Groups

All experiments were carried out after approval by the responsible animal welfare committee of the Landesuntersuchungsamt Rheinland-Pfalz (23177-0/G17-1-062) and according to current national and international guidelines [22, 23]. Forty-three female 8- to 9-week-old C57BL/6N mice (Janvier Labs, Le Genest-Saint-Isle, France) were kept in groups of two or three under standard conditions (12 h light/dark cycle, 22-24°C, 55% humidity) with access to food and water *ad libitum*. Mice were transferred to laboratory facilities five days prior to the start of the experiment and randomly assigned to four different surgical procedure groups. Two groups received an isolated trauma, either controlled cortical impact (CCI, *n* = 12) or fracture of the left femur (FF, *n* = 12), whereas a third group received combined injury (CCI+FF, *n* = 11) and a fourth group was subjected to sham operation (sham, *n* = 8).

### 2.2. Operative Procedures and Analgesic Management

Tramadol (100 mg/ml, Ratiopharm, Ulm, Germany) was added to the drinking water of all experimental groups two days before surgery up to the endpoint of the study at 5 dpi to maintain a sufficient level of analgesia during the postoperative period. All mice received an injection of 50 *μ*g/kg body weight fentanyl (Janssen-Cilag NV, Beerse, Belgium) intraperitoneal (i.p.) 15 minutes before being anesthetized with isoflurane (4 Vol% for 60 s continuing 1.5 Vol% maintenance dose). Spontaneous breathing was maintained throughout the procedure, and reflexes were tested repeatedly to verify adequate depth of anaesthesia. Body temperature was continuously detected via rectal probe and maintained at 37°C using a heating pad (Thermolux, Murrhardt, Germany) during the entire procedure.

For the induction of CCI, mice were fixed in a stereotactic frame and skin was incised longitudinally along the midline in a length of 1 cm. A craniotomy of 4 × 4 mm was then drilled above the right parietal cortex, and the excised bone fragment was flapped laterally with the dura mater remaining intact. The cortical impact was applied using a Benchmark™ Stereotaxic Impactor (Leica Biosystems, Wetzlar, Germany; impactor tip diameter: 3 mm, impact velocity: 6 m/s, impact duration: 200 ms, displacement: 1.5 mm) upon the brain surface. After haemostasis, the cranial bone piece was folded back and craniotomy was sealed with tissue glue (Histoacryl®, Braun). Skin incisions were closed with single button sutures.

For femoral fracture, mice were removed from the stereotactic frame, placed on their right side, and a 1 cm longitudinal midline skin incision was made above the left knee joint. The patella was explored and carefully dislocated medially with the patella tendon remaining intact. A thin intraosseous canal was prepared between the femur condyles with the help of a 25 G cannula. After removal of the cannula, the MouseScrew® was inserted into the femoral bone and fracture was induced by using the three-point fracture device (both RISystem AG, Davos, Switzerland) followed by radiographical control at 35 kV/5 s (Faxitron MX-20, Faxitron Bioptics, LLC, Tucson, Arizona, USA). The patella was repositioned, and skin incision closed. Mice were kept in a neonatal incubator (Babytherm 8000, Draeger, Luebeck, Germany) for two hours before they were returned to their home cages. Sham operation included analgesia, anaesthesia, and skin incisions as described above without further surgical procedures; perioperative parameters as well as duration and anaesthetic management were identical in all groups. Early termination criteria were predefined as ≥20% loss of initial body weight and/or exhibiting signs of severe pain or discomfort in accordance with national and international recommendations, and animals were correspondingly screened several times daily. No animals met the early termination criteria, and the mortality of the study population was 2.32% (one sham mouse deceased).

### 2.3. Behavioral Assessment

To assess neurological and motor impairment, a neurologic severity score (NSS, 0-13 points, higher score indicates greater impairment) including assessment of general behavior, coordination and balancing skills, and motoric impairment and a leg performance test (LPT, 0-6 points, higher score indicates greater impairment) were performed one day before as well as 1, 3, and 5 dpi by an investigator blinded to the surgical procedure. LPT served to assess the function of the lower limbs in detail and to rate three categories—extremity movability, extremity robustness, and mice's general mobility in the cage—each by a 0- to 2-point scoring system. Prior to each scoring episode, mice were placed in an open field test (OFT) arena and video tracked for three minutes (Ethovision XT 14, Noldus Information Technology BV, Wageningen, Netherlands). General locomotor abilities including the travelling distance and mean velocity were examined. Additionally, the expression of anxiety-related behavior (AEB) was analyzed separately by registration of rearing and stretch-attend posture (SAP) [24, 25]. Rearing, an exploratory behavior suppressed by anxiety, was registered when a mouse stood on its hind legs. SAP, which is generally associated with anxiety, was registered when a mouse lowered its back and elongated its body [26, 27].

### 2.4. Histology and Immunohistochemistry

Mice were deeply anesthetized with isoflurane at 4 Vol% for 90 s and euthanized by decapitation at 5 dpi. Brains were removed carefully, frozen in powdered dry ice, and stored at -20°C. Brains were cut to 12 *μ*m coronal slices at 16 consecutive brain levels each 500 *μ*m apart starting at bregma +3.14 mm using a cryotome (Cryo-Star NX70, Thermo Fisher Scientific), collected on Superfrost® Plus Slides (Thermo Fisher Scientific Inc., Waltham, MA, USA) and stained with cresyl violet. Images of each section were taken with a camera-equipped stereo microscope (Stemi 305, Zeiss, Oberkochen, Germany), and the areas of hemispheres and lesion, latter defined as absence of stained cell bodies, were delineated using the digital imaging software ZEN (Zeiss). The corresponding volumes were calculated by adding the respective areas of all 16 sections and multiplying the resulting sum by 500 *μ*m. The width of the granular cell layer (GCL) of the dentate gyrus was determined in three predefined locations along the suprapyramidal blade in ipsi- and contralateral hemispheres for each group. Mean values were calculated from two sections (bregma -1.8 to -2.0 mm) of each animal and expressed as ipsilateral/contralateral ratio.

For immunohistochemistry, sections were air dried, fixed in 4% paraformaldehyde in phosphate-buffered saline (PBS), and incubated with blocking solution (5% normal goat serum, 0.5% bovine serum, and 0.1% Triton-X100 in PBS) for 1 h at room temperature (RT). Primary antibodies (rat anti-GFAP Thermo Fisher Scientific Cat# 13-0300, 1 : 500; rabbit anti-Iba-1 WAKO Cat# 019-19741, 1 : 1000) were diluted and applied in blocking solution and incubated at 4°C overnight. The following day, sections were washed in PBS, incubated with secondary antibodies (goat anti-rat IgG (H+L) cross-adsorbed secondary antibody, Alexa Fluor 488 Thermo Fisher Scientific, Cat# A-11006, 1 : 500; goat anti-rabbit IgG (H+L) cross-adsorbed secondary antibody, Alexa Fluor 568 Thermo Fisher Scientific, Cat# A-11011, 1 : 500) in blocking solution for 2 h at RT, counterstained with 4′,6-diamidino-2-phenylindol (DAPI, Sigma), and mounted in ImmunoMount (Thermo Fisher). Images of the perilesional lateral cortex and corresponding regions in the contralateral hemispheres were captured from two brain sections (bregma -1.86 mm to -2.86 mm) of each animal using a laser scanning microscope (LSM510, 20x objective, Zeiss) and identical filter and acquisition parameters. Images were analyzed using ImageJ software (NIH Image), threshold settings, and the “Analyze Particle” plugin by an investigator blinded to the study groups essentially as described [28].

### 2.5. Gene Expression Analysis

Brain tissue samples containing the lesion and perilesional regions or corresponding areas from FF or sham mice were collected from coronal sections (bregma +0.64 mm to -2.86 mm) during histological processing, snap frozen in liquid nitrogen, and stored at -80°C [29]. RNA extraction and cDNA synthesis were performed using RNeasy Kit and QuantiTect Reverse Transcription Kits (both Qiagen) according to the manufacturer's instruction [30]. Gene target sequences were amplified and examined by qRT-PCR (LightCycler 481, Roche) using Absolute Blue qPCR SYBR Green Mix Plus ROX Vial (Thermo Fisher Scientific) or LightCycler® 480 Probes Master (Roche Molecular Systems Inc., Pleasanton, CA, USA). Samples were analyzed in duplicates, and quantification was performed using a target specific standard curve and normalization to the reference gene cyclophilin A (*Ppia*). Sequences of applied primer pairs (5′-3′) are as follows: *C1qa*: fw-CGGGTCTCAAAGGAGAGAGA, rev-TATTGCCTGGATTGCCTTTC; *C2*: fw-CGGTGGTAATTTCACCCTCAG, rev-GGTGTGATGTGAGCTAGACCT; *C3*: fw-CCAGCTCCCCATTAGCTCTG, rev-GCACTTGCCTCTTTAGGAAGTC; *Fos*: fw-CGGGTTTCAACGCCGACTA, rev-TGGCACTAGAGACGGACAGAT; *Gfap*: fw-CGGAGACGCATCACCTCTG, rev-TGGAGGAGTCATTCGAGACAA; *Il1b*: fw-GTGCTGTCGGACCCATATGAG, rev-CAGGAAGAAGGCTTGTGCTC; *Grn*: fw-ATGCTGTGTGCTGTGAGGAC, rev-CACTCCACATTCCCAACCTT; *Mrc1*: fw-GGCTGATTACGAGCAGTGGA, rev-ATGCCAGGGTCACCTTTCAG; *Ppia*: fw-GCGTCTSCTTCGAGCTGTT, rev-RAAGTCACCACCCTGGCA; *Spp1*: fw-ATGTCATCCCTGTTGCCCAG, rev-ATGTCATCCCTGTTGCCCAG; and *Tnfa*: fw-TCTCATCAGTTCTATGGC, rev-GGGAGTAGACAAGGTACAAC.

50 mg femoral bone tissue from seven mice of each study group was snap frozen in liquid nitrogen and grinded in mortars. The grinded tissue was dissolved in 1 ml TRIzol, and RNA was isolated using a standard phenol/chloroform extraction protocol (Ambion Thermo Fisher, Waltham, USA). 1 *μ*g of total RNA was reverse transcribed into cDNA using dNTPs (4you4 dNTPs Mix (10 mM), BIORON GmbH, Ludwigshafen), random primers (Promega, Madison, WI, USA), and MuLV RT (M-MuLV Reverse Transcriptase, M0253S New England Biolabs, Ipswich, USA) according to the manufacturer's instructions. For gene expression analyses, cDNA template underwent PCR amplification (40 cycles) using the SYBR Green (PowerUp™ SYBR® green master mix, Applied Biosystems, Foster City, USA) and sequence specific primers. GAPDH served as a reference gene. Sample amplification was performed with the qTower3 (Jena Analytik, Jena, Germany). There was an initial activation step at 95°C for 2 min followed by denaturation and enzyme activation at 95°C for 15 s and 40 cycles at 60°C for 15 s for annealing and extension. Results were calculated using the 2-*ΔΔ*Ct method, and gene expression levels are presented relative to gene expression of the sham group [31]. Sequences of applied primer pairs (5′-3′) are as follows: *Alpl*: fw-CATCTGCGTCTACTTGGTGC, rev-CACCCCGCTATTCCAAACAG; *Bdnf*: fw-TGCGGATATTGCGAAGGGTT, rev-CACCTGGTGGAACATTGTGG; *Ibsp*: fw-GGACTGCCGAAAGGAAGGTT, rev-GGCCGGTACTTAAAGACCCC; *Gapdh*: fw-ACCCAGAAGACTGTGGATGG, rev-CACATTGGGGGTAGGAACAC; *Grn*: fw-CTGCCCGTTCTCTAAGGGTG; rev-ATCCCCACGAACCATCAACC; *Mrc1*: fw-GTGGAGTGATGGAACCCCAG, rev-CTGTCCGCCCAGTATCCATC; *Runx2*: fw-CCTCGCTCTCTGTTCCTTCT, rev-CATCTGCGTCTACTTGGTGC; and *Spp1*: fw-CCAGCCAAGGACTAACTACGA, rev-AAAGCTTCTCCTCTGAGCTGC.

### 2.6. Immunoblotting

Immunoblotting was carried out essentially as described [32]. Briefly, brain tissue samples of the injured region were homogenized in radioimmunoprecipitation assay (RIPA) buffer (50 mM Tris-HCl, pH 7.5, 150 mM NaCl, 1 mM EDTA, 1% (*v*/*v*) NP-40, 0.1% (*v*/*v*) sodium dodecyl sulfate, complete protease inhibitors (Roche)). Identical amounts of protein (50 *μ*g/sample) were separated by 8% SDS-PAGE and blotted to nitrocellulose membranes. Membranes were blocked in 2.5% skim milk in Tris-buffered saline (TBS) for one hour at RT and incubated with primary antibodies rabbit anti-GFAP (1 : 10,000, DAKO) and mouse anti-GAPDH (1 : 1000, Acris) overnight at 4°C. Subsequently, membranes were washed in TBS+0.1% Tween 20 (TBST) and incubated with secondary goat anti-rabbit or goat anti-mouse IRDye antibodies (Li-Cor, 1 : 15,000) for 1 h at RT. Protein bands were revealed using Odyssey SA Imaging Systems, and signal intensities were quantified by Image Studio Vision 3.1 (both Li-Cor Bioscience, Lincoln, NE, USA) and normalized to the reference protein GAPDH.

### 2.7. Enzyme-Linked Immunosorbent Assay (ELISA)

Blood samples were taken after euthanasia and decapitation, supplemented with 80 *μ*l of heparin sodium (5000 I.U./ml, Ratiopharm, Ulm, Germany), and centrifuged for 8 min at 3800 rpm, and the extracted plasma was stored at -80°C. Plasma samples were diluted 1 : 200, and the concentrations of osteopontin and progranulin were analyzed using Quantikine Mouse/Rat Osteopontin Immunoassay and Quantikine Mouse Progranulin Immunoassay (both R&D Systems, Inc., Minneapolis, MN, USA) according to the manufacturer's instructions. The absorbance was determined at 450 nm using a microplate reader (MRX TC II Microplate Absorbance Reader, Dynex Technologies, Chantilly, VA, USA), and the osteopontin and progranulin concentrations were expressed as ng/ml.

### 2.8. Statistical Analysis

All data were analyzed using GraphPad Prism software (version 8, GraphPad Software Inc., San Diego, California, USA). Outliers were identified using Rout's test and excluded from further evaluation. Data distribution was analyzed by Shapiro-Wilk normality test and QQ plots and comparisons between two groups were calculated by unpaired Student's *t*-test for parametric data or Mann–Whitney *U* test for nonparametric data. Comparative analysis of more than two groups was performed by ordinary one-way analysis of variance (ANOVA) or Kruskal-Wallis test followed by Holm-Sidak's or Dunn's multiple comparison test depending on data distribution. Experimental responses of more than two groups evaluated at multiple time points, in specific behavioral testing and body weight measurements, have been calculated using two-way ANOVA followed by Holm-Sidak's or Tukey's multiple comparison test. Values are presented as mean ± standard error of the mean (SEM); ^∗^*p* < 0.05, ^∗∗^*p* < 0.01, and ^∗∗∗^*p* < 0.001.

## 3. Results

### 3.1. Femoral Fracture Impairs Behavioral Outcome after TBI

**Figure 1:**
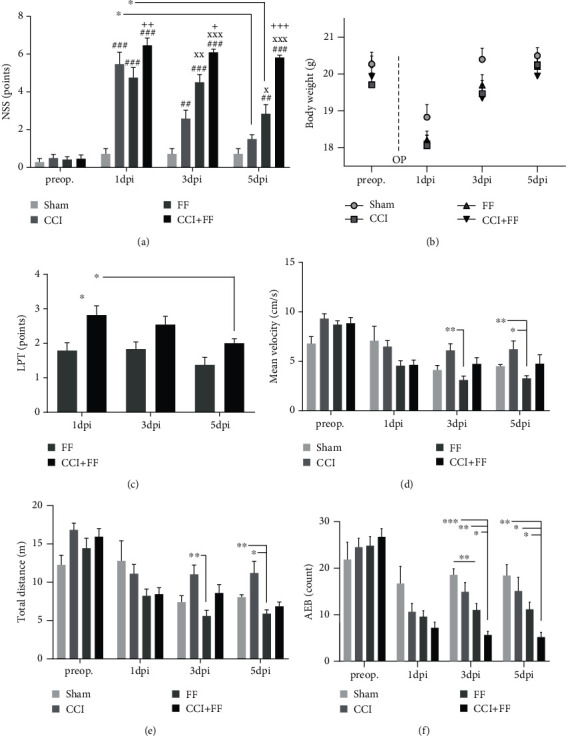
Femoral fracture impairs behavioral outcome after TBI. (a) NSS one day before as well as 1, 3, and 5 dpi. CCI mice showed an increased NSS compared to sham at 1 and 3 dpi and almost completely recovered until 5 dpi. Animals with FF demonstrated greater impairments in relation to sham up to 5 dpi and recovered from 1 to 5 dpi. CCI+FF mice showed an increased NSS from 1 to 5 dpi and did not recover over time. Significance indicators: ^#^significant compared to sham, ^+^significant compared to FF, ^x^significant compared to CCI, ^∗^significant recovery from 1 dpi to 5 dpi. (b) Body weight one day before operation (preop.) and 1, 3, and 5 dpi was not significantly altered between the groups. (c) Leg performance test (LPT), performed at 1, 3, and 5 dpi in the FF and CCI+FF groups. CCI+FF mice showed higher scores at 1 dpi and recovered from 1 to 5 dpi. (d, e) Mean velocity and total distance in the OFT one day before and 1, 3, and 5 dpi. FF mice showed less locomotion in comparison to CCI 3 dpi and CCI and sham 5 dpi. CCI+FF mice did not differ from other experimental groups. (f) Anxiety expression behavior (AEB) one day before and 1, 3, and 5 dpi. CCI+FF mice showed reduced AEB compared to all other groups at 3 and 5 dpi. Values of all data represent mean ± SEM; *p* values were calculated dependent on data distribution by Tukey's multiple comparison test (a, d, e) or Holm-Sidak's multiple comparison test (b, c, f).

Neurological deficits were evaluated using a neurological severity score (NSS, 0-13 pt). Mice subjected to CCI or FF showed an increased NSS at 1 dpi and 3 dpi compared to sham animals. Mice of the combined injury group (CCI+FF) exhibited more severe deficits compared to single trauma, CCI or FF, at 3 dpi and 5 dpi (Figure 1(a)). Accordingly, we observed significant recovery from 1 dpi to 5 dpi both in the CCI and FF groups but sustained impairment in animals with CCI+FF. No significant differences were observed in the animal's body weight (Figure 1(b)).

As neurological assessment is partially based on motoric function, a leg performance test (LPT, 0-6 pt) was carried out to delineate impairments caused by the femoral fracture, focusing on mobility and physical capacity of the lower limbs. LPT, which has been executed in the FF and CCI+FF groups, demonstrated a reduced mobility and physical capacity of the lower limbs in CCI+FF mice, yet a statistically significant difference between these groups was evident at 1 dpi only. CCI+FF mice significantly recovered their leg performance from 1 dpi to 5 dpi, whereas this effect was not detectable in animals with single FF indicating that TBI aggravated leg performance (Figure 1(c)).

To further unveil impairments caused by CCI and/or FF, general locomotor abilities (i.e., total travelling distance and mean velocity) were assessed in the OFT. CCI+FF animals showed no impairments in comparison to other study groups until the study endpoint at 5 dpi (Figures 1(d) and 1(e)). Anxiety-related behavior was assessed by quantitative registration of rearing and supported rearing and stretch-attend posture (SAP) [33], and both behavioral parameters were compiled as AEB [25]. We noticed reduced rearing in the CCI+FF and FF groups but increased SAP in FF mice compared to sham at 5 dpi. CCI+FF animals displayed significantly reduced AEB compared to all other study groups at 5 dpi. Interestingly, all animals subjected to trauma induction (CCI and/or FF) exhibited reduced AEB at 1, 3, and 5 dpi when compared to preoperative baseline values (Figure 1(f)).

### 3.2. Femoral Fracture Increases Structural Brain Damage after TBI

**Figure 2:**
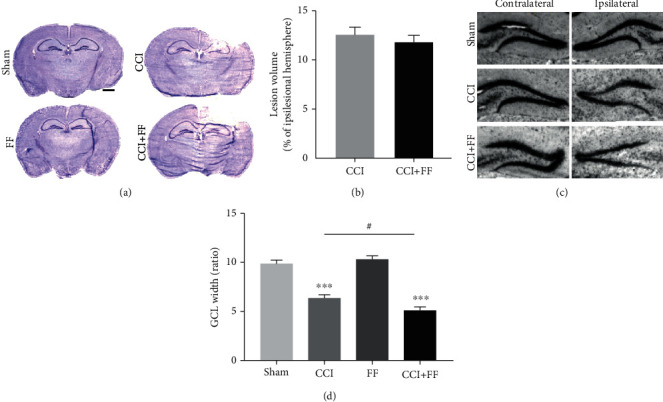
Femoral fracture increases structural brain damage after TBI. (a) Representative images of cresyl violet-stained brain sections, bregma -2.00 mm. Scale bar: 1 mm. (b) Lesion volume expressed as percentage of the ipsilesional hemisphere was not significantly altered in CCI versus CCI+FF mice. (c) Representative images of ipsi- and contralateral dentate gyrus of sham, CCI, and CCI+FF mice, bregma -2.00 mm, showing substance loss in the granular cell layer (GCL). (d) GCL width was determined in the suprapyramidal blade and calculated as ipsi- to contralateral ratio. CCI and CCI+FF showed reduced ratios compared to sham and FF, indicating a posttraumatic substance loss of ipsilesional GCL after CCI. CCI+FF showed significant reduction of GCL width compared to isolated CCI. ^∗^Differences of CCI or CCI+FF compared to FF or sham, ^#^differences between CCI and CCI+FF. Values represent mean ± SEM; *p* values were calculated by Student's *t*-test (b) and Holm-Sidak's multiple comparison test (d).

Cresyl violet staining of brain sections followed by lesion volumetry revealed the extent of the cerebral injury at 5 dpi, involving cortical and subcortical regions including the dorsal hippocampus (Figure 2(a)). Lesion volume in mice with single CCI injury did not differ compared to combined CCI+FF injury (Figure 2(b)). To examine brain damage in more distant locations, width measurements of the dentate gyrus GCL were performed (Figure 2(c)). Calculating the ratios of the ipsi- to contralateral GCL widths of the suprapyramidal blade resulted in a significantly decreased ratio in CCI+FF mice compared to isolated CCI, indicating an increased loss of substance in this particular brain region (Figure 2(d)).

### 3.3. Femoral Fracture Does Not Influence TBI-Induced Gene Expression in the Brain

**Figure 3:**
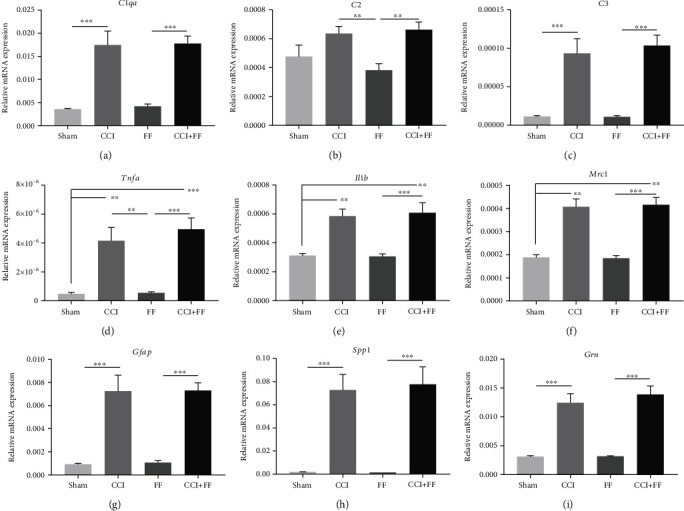
Femoral fracture does not influence TBI-induced gene expression in the brain. (a–i) Gene expression analyses for brain inflammatory markers in perilesional brain tissue normalized to PPIA at 5 dpi. mRNA expressions of all examined genes were increased in CCI and CCI+FF mice compared to sham and FF. Gene expression in CCI and CCI+FF was not significantly different. Values represent mean ± SEM; *p* values were calculated by Holm-Sidak's multiple comparison test or by Dunn's multiple comparison test (*Mrc1*, f).

To evaluate brain inflammation in response to injury, we performed gene expression analyses by qPCR for a panel of markers comprising genes coding for the complement factors C1QA (Figure 3(a), *C1qa*), C2 (Figure 3(b), *C2*), and C3 (Figure 3(c), *C3*), the proinflammatory cytokines TNF*α* (Figure 3(d), *Tnfa*) and IL-1*β* (Figure 3(e), *Il1b*), the microglia/macrophage marker MRC1/CD206 (Figure 3(f), *Mrc1*), and the astrocyte activation marker GFAP (Figure 3(g), *Gfap*). Additionally, gene expression of osteopontin (Figure 3(h), *Spp1*) and progranulin (Figure 3(i), *Grn*) was analyzed. The expression of all gene markers was strongly upregulated in the injured brain tissue of CCI and polytrauma mice (CCI+FF) compared to FF or sham mice. No differences were observed between CCI and CCI+FF, indicating that concomitant FF does not influence the TBI-induced gene expression of inflammatory markers in the brain.

### 3.4. Astrogliosis Is Locally Increased after TBI by Concomitant Femoral Fracture

**Figure 4:**
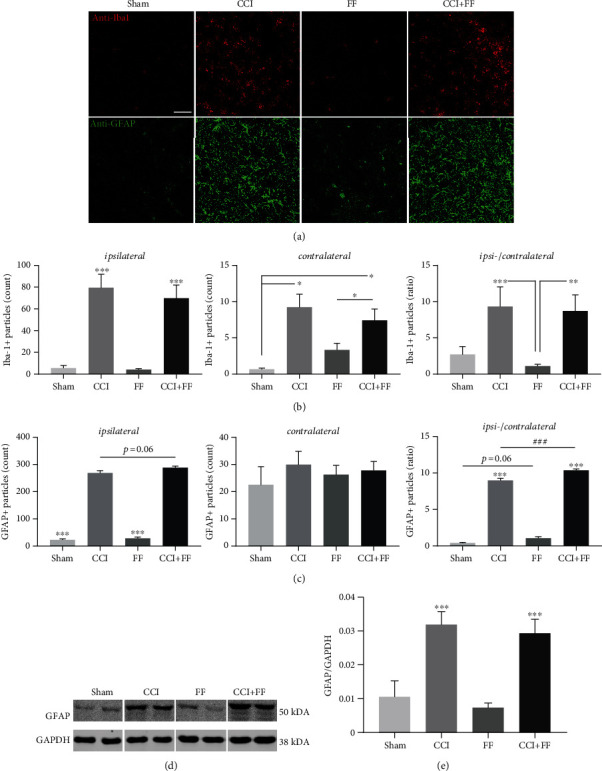
Astrogliosis is locally increased after TBI by concomitant femoral fracture. (a) Representative immunofluorescence images of brain sections at 5 dpi showing microglia/macrophages (anti-Iba1, red) and reactive astrocytes (anti-GFAP, green) in the perilesional cortex of CCI and CCI+FF mice and corresponding regions of FF and sham mice (bregma -2.00 mm, scale bar: 50 *μ*m). Quantification of (b) Iba-1 or (c) GFAP immunopositive particles in ipsi- and corresponding contralateral areas and the calculated ipsi-/contralateral ratios. The ipsi-/contralateral ratio of GFAP immunopositive particles was significantly increased in CCI+FF versus CCI (^###^, c). Isolated FF showed a statistical trend towards an increased ipsi-/contralateral ratio in comparison to sham (*p* = 0.06). (d) Representative immunoblot demonstrating TBI-induced GFAP protein expression in the ipsilesional brain tissue at 5 dpi in CCI and CCI+FF mice. GAPDH served as reference protein. (e) Quantification of protein band intensities of GFAP normalized to GAPDH. Expression was increased in CCI and CCI+FF mice in comparison to sham and FF (^∗∗∗^) but not significantly altered in CCI versus CCI+FF groups. Values represent mean ± SEM; *p* values were calculated by Holm-Sidak's multiple comparison test.

To study local glial activation the injured brain, we performed double immunostaining with antibodies specific to the astrocyte marker GFAP and the microglia/macrophage marker Iba-1 (Figure 4(a)). Images were acquired at the perilesional cortex lateral from the lesion site and corresponding regions in contralesional hemispheres or noninjured regions in FF and sham mice and processed for digital quantification. CCI and CCI+FF mice showed strongly increased numbers of GFAP and Iba-1 immunopositive structures compared to FF and sham groups. However, combined injury did not affect their number or distribution compared to isolated CCI (Figure 4(b)). Counts of GFAP immunopositive structures in the perilesional area showed a trend towards increased GFAP expression after CCI+FF compared to CCI alone (*p* = 0.06, Figure 4(c)). Calculating the ratio between ipsi- and corresponding contralateral hemispheres revealed a significantly higher ratio in CCI+FF mice compared to CCI. Interestingly, mice with single FF also showed a trend towards increased GFAP ratios in comparison to sham (*p* = 0.06, Figure 4(c)), suggesting that an isolated femoral fracture may represent an activating factor for cerebral astrocytes. However, immunoblotting, to reveal total GFAP protein levels in the ipsilesional brain tissue, also resulted in about threefold higher levels for mice of the CCI and CCI+FF groups in comparison to sham and FF but no significant differences were identified for total GFAP levels between animals with single cerebral and combined trauma (Figures 4(d) and 4(e)).

### 3.5. Concomitant TBI Alters Gene Expression in the Fractured Femur

**Figure 5:**
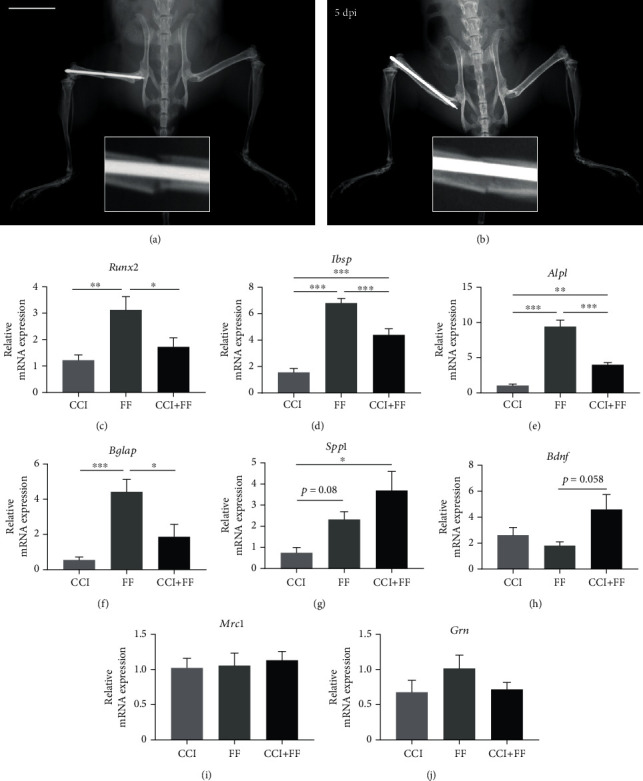
Concomitant TBI alters gene expression in the fractured femur. Representative X-ray images of the osteosynthetic stabilized femoral fracture during (a) surgical procedure and (b) 5 dpi *post mortem*, fracture gaps are enlarged for detailed view. Scale bar: 1 cm. (c–j) Gene expression analyses in the perifractural bone tissue normalized to GAPDH and to sham 5 dpi (*n* = 7/group). Gene expression of Runx2 (*Runx2*, c), bone sialoprotein (*Ibsp*, d), alkaline phosphatase protein (*Alpl*, e), and osteocalcin (*Bglap*, f) was increased in fractured bones of the FF group compared to uninjured femora of the isolated CCI group, but significantly reduced by the additional CCI in CCI+FF mice. Gene expression of osteopontin (*Spp1*, g) was increased in CCI+FF mice compared to sham. Gene expression of BDNF (*Bdnf*, h) was increased in CCI+FF compared to isolated CCI but the differences failed statistical significance (*p* = 0.058). No significant differences were revealed for the gene expressions of MRC1 (*Mrc1*, i) and progranulin (*Grn*, j). Values represent mean ± SEM; *p* values were calculated by Holm-Sidak's multiple comparison test.

Gene expression analyses of bone tissue were performed for several markers of bone turnover and immunological activation [12]. Gene expression of Runx2 (*Runx2*), bone sialoprotein (*Ibsp*), alkaline phosphatase (*Alpl*), and osteocalcin (*Bglap*) was significantly decreased in mice of the polytrauma (CCI+FF) group compared to those with isolated femoral fracture (Figures 5(c)–5(f)). Expression of osteopontin (*Spp1*) was slightly increased in mice with isolated FF compared to CCI (*p* = 0.08) and was significantly increased in polytrauma (CCI+FF) mice in comparison to isolated CCI (Figure 5(g)). For BDNF, a trend towards increased expression was found in CCI+FF in comparison to FF groups (*p* = 0.06) but not in comparison to isolated CCI (Figure 5(h)). Gene expressions of MRC1 (*Mrc1*) and progranulin (*Grn*) were not altered (Figures 5(i) and 5(j)).

### 3.6. Combined Injury Increases Plasma Levels of the Inflammation and Repair Biomarkers Osteopontin and Progranulin

**Figure 6:**
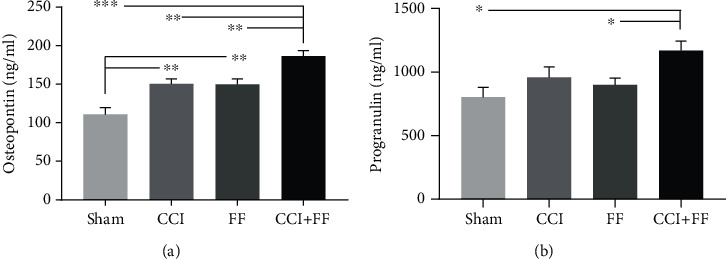
Combined injury increases plasma levels of the inflammatory and repair biomarkers osteopontin and progranulin. (a, b) Osteopontin and progranulin protein plasma concentrations as determined by ELISA at 5 dpi. Osteopontin was increased in CCI+FF mice compared to all other groups and in the CCI and FF groups in comparison to sham. Progranulin was increased in CCI+FF mice in comparison to sham and FF. Values represent mean ± SEM; *p* values were calculated using Holm-Sidak's multiple comparison test.

Finally, we asked whether circulating biomarkers can distinguish between single TBI, or femoral fracture, and concomitant TBI and femoral fracture injuries. To this end, we determined plasma levels of the inflammation and repair markers osteopontin [34, 35] and progranulin [36, 37] at 5 dpi (Figure 6). We found increased plasma levels of osteopontin in mice subjected to CCI or FF alone or CCI+FF compared to sham. Moreover, plasma osteopontin concentration was significantly elevated following combined injury compared to single injuries suggesting an additive effect of the combined injury (Figure 6(a)). Progranulin plasma levels were increased in CCI+FF compared to sham and FF groups but not in comparison to CCI (Figure 6(b)). Thus, plasma concentrations of osteopontin and progranulin were increased in response to injury but only osteopontin was significantly increased in TBI with concomitant femoral fracture.

## 4. Discussion

This study investigated reciprocal effects between TBI (CCI) and femoral fracture (FF) in mice. Combined injuries showed the strongest neuromotor impairment over time and failure of neuromotor recovery compared to isolated TBI or FF. The negative effects of FF on neuromotor performance in the present study are in agreement with previous findings in animals with combined brain and leg bone injury [20, 21, 38]. It should be noted that the majority of tasks designed to evaluate neurological deficits, including those applied in the present study, involve locomotion and therefore limb fracture can represent a confounding factor. To elucidate its potential influence in our study, the mobility and performance of the lower limbs and general locomotor abilities were assessed separately by LPT and OFT. Although both assessments revealed slight motoric frailty caused by the femoral trauma, this observation did not explain the persisting neuromotor impairment in the polytrauma group. It appears rather plausible that additive effects of TBI and FF account for severe neuromotor impairment and failure of neuromotor recovery. To capture more complex behavioral alterations, we evaluated the anxiety-related frequencies of rearing and stretch-attend postures. Interestingly, mice undergoing combined or single traumatic injury showed decreased anxiety-like behavior at 1 dpi and 3 dpi compared to the preoperative time point and this behavioral abnormality persisted until the endpoint of our study at 5 dpi in CCI+FF mice. Decreased anxiety-like behavior has been described in the murine CCI model of TBI [39, 40], and less risk-taking behavior was reported after tibial fracture in mice [41]. However, only scarce data exist in this context and more research is needed to explore how FF modifies anxiety-like behavior in combination with TBI.

In the present study, the overall brain lesion volume was not different between mice with isolated TBI and concomitant FF. However, in the perilesional GCL of the hippocampus, an increased substance loss was found in polytrauma mice. Histopathological aggravations after TBI, when accompanied by an osseous trauma, have been demonstrated in previous studies [20, 21, 38, 42]. Two of these studies demonstrated increased mRNA expression and brain tissue concentration of the proinflammatory cytokine IL-1*β* at early (24 h and 4 d) and late (35 d) posttraumatic time points, respectively [20, 21]. It was also shown that administration of an IL-1 receptor antagonist (ra) attenuated neuroinflammation in a mouse model of TBI and concomitant tibial fracture [43]. These results suggest that anti-inflammatory treatment could be a therapeutic option for polytrauma. Importantly, IL-1ra is currently being investigated in a clinical dose range study for moderate to severe TBI (ClinicalTrials.gov Identifier: NCT02997371).

In the present study, gene expression analyses and immunohistochemistry confirmed robust activation of microglia and astrocytes at 5 dpi, but gene expression of the acute phase cytokines TNF*α* and IL-1*β*, gene markers of the activated complement system, or the reactive astrocyte marker GFAP, was not different between combined and single injury groups. However, it is possible that inflammatory peaks in polytrauma mice may have preceded our observation time point at 5 dpi. Nevertheless, immunostainings at perilesional sites and corresponding contralateral sites revealed an increased ratio of reactive astrocytes in polytrauma compared to isolated TBI, which is in agreement with previous studies [20, 38]. A trend towards increased ipsi- to contralateral hemispheric ratio was also observed in the uninjured brains of animals with isolated femoral fracture compared to sham. Indeed, extremity bone fracture was reported to cause central sensorimotor dysfunction in patients [44] and increased proinflammatory cytokine levels were found in the hippocampus after tibial fracture [45]. These findings support the existence of retrograde effects resulting from FF, possibly reflected by an increased reactivity of astrocytes in our model.

An important finding of this study is that TBI attenuated increased gene expression of the early osteoblast differentiation and bone formation markers Runx2, alkaline phosphatase, bone sialoprotein, and osteocalcin in the injured bone at 5 dpi. Interestingly, recent data showed decreased gene expression of Runx2 and osteocalcin in uninjured bones at 8 weeks after TBI [46] supporting the hypothesis that brain injury-derived factors, possibly inflammatory mediators, alter gene transcription in bones. Indeed, Runx2 is widely regarded as a key transcription factor of osteoblast differentiation and bone formation and delayed fracture healing has been shown in Runx2 mouse mutants [47, 48]. Most existing data from murine polytrauma models resulted from *μ*CT and density measurement of bone formation and callus size and focused on later posttraumatic time points [14–16]. Early gene expression regulation in the damaged osseous tissue is a less investigated research field. Currently, increased callus volumes are predominantly described in fractures combined with TBI, mostly referring to time frames up to 4 weeks post injury [15, 16, 49]. On the other hand, findings in a closed head TBI and tibial fracture model revealed fading effects on callus volume at later investigation points [14]. In contrast, another study reported detrimental effects of TBI on healthy bone formation just as fracture location itself seems to be an issue of significance [50, 51]. However, the suppression of Runx2 and bone matrix genes in the present study appears unexpected in the light of the reported data so far. Given the scarce data on early bone healing after TBI, further investigations in appropriate animal models are required. Interestingly, we noted increased gene expression of BDNF and osteopontin in polytrauma mice compared to isolated femoral fracture. Promoting effects of BDNF on osteogenesis of human bone mesenchymal stem cells have been shown before and represent an alternate pathway interfering with callus formation [52].

With two coexisting traumatic regions of interest, circulating immune cells and released factors may affect both injury sites. We analyzed plasma levels of osteopontin and progranulin. Progranulin is upregulated in response to injury and inflammation [36], confers neuroprotection, and serves as an anti-inflammatory mediator after experimental TBI [28]. In addition, recombinant PRGN enhances bone regeneration and osteoblast differentiation by inhibition of TNF*α* signalling [53, 54]. We found higher levels of plasma PRGN in mice undergoing combined injury in comparison to sham and single FF but not compared to TBI alone. In contrast, osteopontin plasma levels were significantly increased in polytrauma mice compared to groups with isolated TBI or FF. While osteopontin gene expression was not altered in brain tissues of TBI and polytrauma mice, increased expression was revealed in the fractured femoral bone and additive expression was observed in polytraumatized animals. Osteopontin is expressed in many tissues by different cell types and has become a therapeutic target in brain injury models [35]. In vitro studies revealed inhibitory effects on microglial proliferation and migration, but controversial data were reported regarding its neuroprotective properties [55–57]. In the present study, elevated plasma levels of PRGN and OPN were found in combined TBI and femoral fracture. Since anti-inflammatory treatment mitigates neuroinflammation in murine polytrauma [43], PGRN and OPN can represent potential therapeutic targets in polytrauma models and it would be important to test their usefulness as clinical biomarkers.

This study has some limitations which need to be considered. First, only female mice were examined. Sex differences have recently been highlighted in relation to both human TBI and animal models of TBI [58] but not in polytrauma. Future studies are therefore needed to investigate potential sex differences in combined models for TBI and fracture injuries. Another limitation is that the CCI model of TBI requires craniotomy which represents an osseous trauma in addition to the femoral trauma. Therefore, we cannot exclude the possibility that craniotomy influences reciprocal effects between brain and femoral injuries. A closed head injury model (e.g., by weight drop) combined with leg bone fracture may circumvent this limitation and reveal minor interactions that were not found in the current work. Finally, it would be important to allow longer recovery times in our model to examine outcome parameters, i.e., (heterotopic) ossification, neurological outcome, and brain histopathology along with possible alterations in gene and protein markers assessed in the present study.

## 5. Conclusions

The objective of this study was to investigate early reciprocal effects in a murine model of TBI and femoral fracture. While femoral fracture aggravated TBI-induced neurological and behavioral impairments and increased local brain histopathology, TBI attenuated fracture-induced upregulation of bone healing-associated genes. Combined injury also increased circulating levels of PGRN and OPN, two biomarkers of inflammation and repair, which may exert pleiotropic actions at both traumatic spots. Additional studies to test their utility as biomarkers in clinical settings of polytrauma appear advisable. Furthermore, the present and future findings in this polytrauma model may have implications for therapeutic approaches to interfere with the pathological crosstalk between TBI and concomitant bone fracture.

## Data Availability

The datasets generated and/or analyzed during the current study are available on reasonable request from the corresponding author.

## Abbreviations

AEB: Anxiety expression behavior
ALP: Alkaline phosphatase
ANOVA: Analysis of variance
BBB: Blood-brain barrier
BDNF: Brain-derived neurotrophic factor
BSP: Bone sialoprotein
CCI: Controlled cortical impact
DAPI: 4′,6-Diamidino-2-phenylindole
DG: Dentate gyrus
dpi: Days post injury
EDTA: Ethylenediaminetetraacetic acid
ELISA: Enzyme-linked immunosorbent assay
FF: Femoral fracture
Fig.: Figure
G: Gauge
GAPDH: Glyceraldehyde 3-phosphate dehydrogenase
GCL: Granular cell layer
GFAP: Glial fibrillary acidic protein
Grn: Granulin
h: Hour
HO-1: Heme oxygenase 1
Iba-1: Ionized calcium-binding adapter molecule 1
IHC: Immunohistochemistry
IL-1*β*: Interleukin 1 beta
IL-6: Interleukin 6
kV: Kilovolt
LPT: Leg performance test
min: Minute
MRC1: Mannose receptor C-type 1
NSS: Neurological severity score
OFT: Open field test
PBS: Phosphate-buffered saline
PRGN: Progranulin
PPIA: Peptidylprolyl isomerase A
pt: Point
qRT-PCR: Quantitative real-time polymerase chain reaction
RIPA: Radioimmunoprecipitation assay buffer
ROS: Reactive oxygen species
RT: Room temperature
Runx2: Runt-related transcription factor 2
s: Second
SAP: Stretch-attend posture
SDS-PAGE: Sodium dodecyl sulfate-polyacrylamide gel electrophoresis
SEM: Standard error of the mean
SPP1: Secreted phosphoprotein 1
TBI: Traumatic brain injury
TBS: Tris-buffered saline
TNF*α*: Tumor necrosis factor alpha
YLD: Years lived with disability.
